# Maternal Docosahexaenoic Acid Status during Pregnancy and Its Impact on Infant Neurodevelopment

**DOI:** 10.3390/nu12123615

**Published:** 2020-11-25

**Authors:** Sanjay Basak, Rahul Mallick, Asim K. Duttaroy

**Affiliations:** 1 Molecular Biology Division, National Institute Nutrition, Indian Council of Medical Research, Hyderabad-500 007, India; sba_bioc@yahoo.com; 2 Department of Biotechnology and Molecular Medicine, A.I. Virtanen Institute for Molecular Sciences, University of Eastern Finland, 70211 Kuopio, Finland; rahul.mallick@uef.fi; 3 Department of Nutrition, IMB, Faculty of Medicine, University of Oslo, 0317 Oslo, Norway

**Keywords:** DHA, brain, MFSD2a, SPM, fetus, placenta, infant, neurogenesis, pregnancy, pre-term

## Abstract

Dietary components are essential for the structural and functional development of the brain. Among these, docosahexaenoic acid, 22:6n-3 (DHA), is critically necessary for the structure and development of the growing fetal brain *in utero*. DHA is the major n-3 long-chain polyunsaturated fatty acid in brain gray matter representing about 15% of all fatty acids in the human frontal cortex. DHA affects neurogenesis, neurotransmitter, synaptic plasticity and transmission, and signal transduction in the brain. Data from human and animal studies suggest that adequate levels of DHA in neural membranes are required for maturation of cortical astrocyte, neurovascular coupling, and glucose uptake and metabolism. Besides, some metabolites of DHA protect from oxidative tissue injury and stress in the brain. A low DHA level in the brain results in behavioral changes and is associated with learning difficulties and dementia. In humans, the third trimester-placental supply of maternal DHA to the growing fetus is critically important as the growing brain obligatory requires DHA during this window period. Besides, DHA is also involved in the early placentation process, essential for placental development. This underscores the importance of maternal intake of DHA for the structural and functional development of the brain. This review describes DHA’s multiple roles during gestation, lactation, and the consequences of its lower intake during pregnancy and postnatally on the 2019 brain development and function.

## 1. Introduction

Docosahexaenoic acid, 22:6n-3(DHA), an n-3 (omega-3) long-chain polyunsaturated fatty acid (LCPUFA), plays various essential roles in human health [1,2,3,4]. DHA takes part in several biological processes, such as angiogenesis, immune modulation, inflammatory response, signal transduction, apoptosis, cell proliferation, and a host of the membrane, cellular and molecular functions that affect health and diseases [3,5,6]. The DHA metabolites also mediate their roles in cellular signaling processes [7]. Inadequate dietary consumption of DHA and eicosapentaenoic acid, 22:5n-3 (EPA) impairs the optimal growth of the feto-placental unit and imposes plausible risks of cognitive decline, inflammatory disease, cardiovascular disease, inflammatory disorders, behavioral changes, and mental stress in later life [5,8,9]. While the metabolites of EPA and DHA, such as eicosanoids and docosanoids, are involved in cell signaling, DHA is primarily used for membrane structure and function [3,4]. Modern refined diets are mostly deficient in n-3 PUFAs, leading to sub-optimal organ function that may predispose individuals to an increased risk of diseases [9,10,11].

The recommended intake of DHA and the EPA is based on the quantum of data supporting beneficial outcomes in protecting cognitive development and cardiovascular diseases. Experts are agreed upon to enhance n-3 LCPUFA intake via increased seafood or supplementary approaches. An n-3 LCPUFA intake of no less than 250–500 mg/d should be made available to maintain healthy adults’ physiological needs in their daily routine. Even though DHA consumption during pregnancy is an essential consideration as it is required for fetal brain development and growth, a limited number of countries have adopted and implemented appropriate guidelines for the n-3 LCPUFA consumption during pregnancy. DHA’s requirement during placental growth and early development has been highlighted recently. Thus, low maternal DHA status may lead to the placenta’s functional inadequacy and accordingly alter fetoplacental growth and development. Therefore, DHA supplementation well before gestation can be considered to prevent feto-placental associated developmental disorders.

Several comprehensive articles have focused on DHA and its impacts on human health [3,4,12,13,14]. However, this review has primarily concentrated on maternal DHA status during pregnancy on neurocognitive development in infants.

## 2. DHA and Its Metabolites: Effects on the Structure and Function of the Human Brain

N-3 PUFAs have essential functions on human health, and various studies have explained the molecular mechanisms underlying the effects of DHA in various tissues, including the brain. DHA is the predominant n-3 LCPUFA within the brain. Several neurophysiological functions are attributed to DHA, including the cell-survival, neuroinflammation, neurogenesis cellular signaling, and its protective function in maintaining blood-brain barrier integrity. Because of these vital roles of DHA in the brain, any alteration of DHA metabolism in the brain, as a cause or consequence, affects several neurological and psychiatric conditions.

DHA derivatives include the families of specialized pro-resolving mediators (SPMs), such as lipoxins, resolvins (resolvin D (RvD), and resolvin E (RvE)), protectins, and maresins, are known to have diverse biological activities [15,16]. SPMs actively thwarted the inflammatory response by stimulating specific G-protein-coupled receptors (GPR) expressed on immune cells that propagate both the anti-inflammatory and pro-resolving processes [15,16]. The anti-inflammatory activities of SPMs are mediated by inflammatory scavenging interleukins (IL) such as IL-10, IL-1 decoy receptors, and IL-1 receptor antagonists and anti-inflammatory cytokines [15,16]. The SPMs activate their actions by several mechanisms that include the anti-inflammatory resolution, blocking the intracellular pathways leading to inflammation, downregulation of pro-inflammatory cytokines, and clearance of inflammatory cell debris macrophages, as well as stabilization of immune cells counts to basal levels [15,16]. The importance of SPMs in the inflammatory resolution is widely reported in chronic pathologies, including brain inflammation when their production remained insufficient, and the administration of SPMs exogenously reduces the inflammatory process and protects inflamed tissue(s) [15,17]. Dietary supplementation with metabolic precursors of SPMs can increase their availability and resolve the inflammatory process following neurological injury.

In the brain, DHA and arachidonic acid, 20:4n-6 (ARA) are incorporated into cell membrane phospholipids and thus influence their metabolism. ARA represents approximately 20% of the total amount of fatty acids in neurons. ARA, primarily esterified in phospholipids of the membranes, is released by the phospholipases A_2_. Several enzymes convert free ARA into several eicosanoids, and these metabolites may be involved in neuroinflammation [18]. Unesterified ARA can also directly modify synaptic functions [19]. Therefore, the level of intracellular free ARA plays a critical role in both neuroinflammation and synaptic functions. Inflammation may prolong due to the presence of ARA-derived eicosanoids, such as prostaglandin E_2_ and leukotriene B_4_, whereas n-3-derived eicosanoids decrease the levels of these inflammatory compounds and their activity. Thus, supplementation of n-3 fatty acids can restore or reverse the balance of lipid mediator precursors. Metabolic precursors of the inflammation-resolving SPMs, including DHA itself, having vital inflammation resolution effects in brain injury [20]. Intravenously administered low-dose DHA in rats showed significant tissue-sparing effects in the peri-infarct region of the middle cerebral artery occlusion compared to those who received high or medium DHA doses [21]. DHA-treated rats improved significantly in neurological performance up to 7 days following middle cerebral artery occlusion. Following experimental traumatic brain injury in rats, a DHA-enriched diet for 12 days preserved brain-derived neurotrophic factor (BDNF) concomitant improved learning capability [20]. Although DHA’s mechanism in rescuing neurological injury is not known, its potential therapeutic warrants further investigations.

Resolvins, protectins, and maresins have SPM functions. SPMs are produced from DHA either spontaneously or by aspirin, involving enzymes, such as lipoxygenase (LOX) and acetylated cyclooxygenase-2 (COX-2) enzymatic pathway. Two distinct classes of the resolvins (Rvs) are synthesized from DHA by different biosynthetic paths, denoted as 17S- and 17RD- series resolvins during inflammation of the resolution [22,23]. The strong anti-inflammatory and pro-resolving activities of Rvs were demonstrated in animal models [7]. Rv of E and D series are endogenously produced from EPA and DHA, respectively. Both RvD1 and RvE1 may potentially lower inflammation. Conversion of DHA to 17(S)-hydroxy-containing RvD1–D4 and conjugation to triene-containing docosanoid structures are carried out by LOX [24]. Rvs can reduce the augmented pain by regulating the mediators of inflammation, ion channels with transient receptor potential, and transmission via the spinal cord. RvD_1_ (17(S)-trihydroxy-docosahexaenoic acid) is structurally different from aspirin-triggered RvD_1._ COX-2 promotes the synthesis of 17(R)-trihydroxy-docosahexaenoic acid, followed by the LOX on 13-hydroxyDHA [25]. The transcription of IL-1β is prevented by RvD1 that reduces the infiltration of neutrophils into the brain [23]. Since all Rvs are potent anti-inflammatory agents, dietary supplementation of n-3LCPUFAs is beneficial in the inflammatory situation.

The anti-inflammatory protectin D_1_ (PD1) is also synthesized endogenously from DHA in neuronal tissues and, therefore, termed neuroprotectin (NPD_1_). The internally formed NPD_1_,17(S)-trihydroxy DHA varies from aspirin-triggered NPD_1_; 17(R)-trihydroxy DHA structurally. Protectins are anti-microbial agents. PD_1_ was shown to prevent infiltration PMN both in vivo and in vitro. NPD_1_ effectively prevents damage to several mice tissues, such as the retina, brain, liver, kidney, and fibrosis [26]. The PD1/NPD1 isomer of PDX can prevent both inflammation and atherogenesis [27,28]. Besides its insulin-sensitizing and glucose regulatory actions, PDX can inhibit inflammation via IL-6 synthesis [29,30]. Maresins, MaR (macrophage mediators in resolving inflammation) are endogenously synthesized from DHA by macrophages through 12-LOX, followed by epoxy hydrolase activity. Maresins have both anti-inflammatory and pro-resolving actions [31]. The two structural variants of MaR are MaR 1 and MaR 2. MaR 1 (7*R*,14*S*-dihydroxy-docosa-4*Z*,8*E*,10*E*,12*Z*,16*Z*,19*Z*-hexaenoic acid) is a family of structurally distinct autacoids [32]. Studies on MaR 1 has potent anti-inflammatory activity even at the nano molar range [33].

Resolvins and protectins are produced from EPA and DHA. Both resolvins and protectins have an anti-inflammatory function. While 18R E-series resolvins (RvE1 and RvE2) are generated from EPA, DHA-derived 17S D-series resolvins (RvD1 to D6), PD1, and MaRs are synthesized during the inflammatory resolution. These inflammation preventive mediators are critically involved in resolving inflammation for the healing process. These EPA- and DHA-derived anti-inflammatory compounds inhibit trans-endothelial migration of PMN, suppress dendritic cell migration, reduce leukocyte infiltration [34]. PD1 blocks the synthesis of T cells-derived tumor necrosis factor alpha TNF-α and interferon gamma IFN-γ and induces apoptosis of these cells, implying its role in the down-regulation of Th1-mediated responses. LOX-dependent Th2-skewed human peripheral blood mononuclear cells-derived PD1 may favor the Th2 phenotype [35]. Several studies suggest that both n-3LCPUFAs, DHA, EPA, and their inflammation preventive mediators, such as MaRs, PDs, and Rvs, play vital role in mitigating the inflammatory process and support wound healing. Therefore, these bioactive lipids could be developed to manage and prevent various inflammatory diseases [34].

### 2.1. DHA Accretion, Supplementation, and Fetal Brain Development

DHA is vital for the development of a healthy brain [12,36,37]. Quantitatively in the brain, DHA is considered as the vital fatty acid [38]. In the human brain, the DHA level is 250–300 times higher than EPA. DHA is predominantly found in the phospholipid fraction of the brain grey matter [39]. The phospholipid distribution of n-3 LCPUFA in the brain showed a striking difference between EPA and DHA. DHA is primarily present in phosphatidylethanolamine and phosphatidylserine fractions, whereas EPA is mostly distributed in the phosphatidylinositol fraction of the membrane phospholipids. DHA is the most abundant n-3 fatty acids in the entire nervous system. DHA is critically required in the neuron regeneration and formation of synapse during the fetus’s development and the first two years following birth [40].

Accumulation of DHA in the fetal brain occurs continuously throughout the gestation but is most active during week 29 to week 40. Supplementation of DHA (200 mg DHA/day) during the third trimester of pregnancy prevented the decrease of maternal DHA status [41]. Due to continuum fetal DHA accretion, the nutritional status of maternal DHA during pre-conception, pregnancy, and lactation demands DHA requirements for the brain and retinal development of babies [42,43]. Neonates with higher DHA concentrations in umbilical plasma phospholipids showed a longer gestational length than neonates with a lower concentration of DHA [44]. The pregnant women, supplemented with 600 mg DHA/day before 20 weeks of gestation until delivery, significantly reduced the preterm delivery and low-birth-weight babies [45]. DHA supplementation improves the DHA status both in the mother and her child because of its efficient transfer through the placenta [46], and breast milk [47,48]. DHA supplementation was beneficial for mothers having low consumption of seafoods [49]. DHA supplementation during pregnancy increases the placental transport of n-3 LCPUFAs via increased expression of fatty acid-binding/ transport proteins [50,51]. Consuming DHA (2.2 g DHA/day) and EPA (1.1 g EPA/day), from the 20th week of pregnancy until the partum showed an improved visual and coordination capacity of the children [52]. Similar beneficial effects were obtained after the supplementation of mothers with 500 mg DHA/day during pregnancy, which correlated the high blood DHA levels with the improved cognitive development of 5.5-year-old children [53]. Similarly, daily supplements of fish oil (500 mg DHA + 150 mg EPA), along with 5-methyltetrahydrofolate (400 μg/day), showed improved cognitive development until 6.5 years of age [54]. Higher DHA levels in plasma and breast milk positively correlate the brain’s growth, development, and visual acuity in the neonate [55,56]. These studies demonstrated that DHA supplementing mothers during pregnancy and lactation or baby food fortified with DHA enhanced DHA levels in the infant tissues with improved neurological and visual development [57]. Conversely, a diet low in n-3 LCPUFA during pregnancy and/or lactation may negatively impact the child’s visual and neurological development [58,59].

Infants fed with breast milk (<0.17% of total fatty acid in milk in contrast to optimal 0.3–0.4% DHA) showed a lower amount of DHA in erythrocytes, reduced visual acuity, and delayed language development as compared to infant fed by breast milk containing 0.36% DHA [43,60]. Women who received DHA (600 mg/day) supplementation during pregnancy from <20 weeks until delivery showed a substantial increase in visual acuity, particularly in male newborns. DHA supplementation may be the best predictor for nervous system development [61]. A direct relationship between higher DHA levels in erythrocytes (in mother and children) and the optimal visual and neuronal development of children was reported [62,63,64,65]. Perinatal DHA supplementation was also reported to reduce the risk of lower intelligence quotient (IQ) scores in children from very low-income families [66,67]. Newborn, when supplemented with DHA enriched formulas, improved cortical maturation and visual function [68]. Six-month-old babies who did not receive maternal milk and fed a formula containing egg yolk enriched with DHA (115 mg DHA/100 g food) showed a significant increase in erythrocyte DHA phospholipids and a better visual development measured at 1 year [69].

Supplementation of a minimum of 0.35% DHA formulated foods favors improved brain development until 4 months after delivery, as evaluated by the term’s mental development index [70]. In addition to DHA’s dietary intake, ARA is obligatory for optimal brain and eye development in infants [71]. The child who did not receive maternal milk must be fed with a formula containing DHA and ARA for optimum brain growth and development until 39 months after birth [72] and even 4 years after birth [71]. A study in newborns (*n* = 343) of 1–9-day-olds fed with formula with varying levels of DHA ((control (DHA 0%); 0.32%; 0.64%; 0.96%, with ARA 0.64% in all formulas) until the 12th month showed that babies fed with formulas containing 0.32% DHA showed a significant improvement in cognitive development as compared to control group [73]. An improved problem-solving skills and memory were noticed when human milk supplemented in DHA (32 mg/day) and ARA (31 mg/day) were fed to the preterm infant (birth weight < 1500 g) until 9 weeks after birth [74]. DHA (0.05 g/100 g) and ARA (0.1 g/100 g) fortified feeding to the preterm infant (birth weight > 2000 g) performed a higher index in mental development scores [75]. The term newborn (*n* = 420) supplemented with n-3 LCPUFA (60 mg of EPA and 250 mg of DHA daily) for six months following birth showed a significant increase of DHA deposition in the erythrocyte phospholipids and language and communication skills development [76]. A follow-up study with a formula (0.5% DHA) feeding preterm children (*n* = 107) from their birth until nine months showed a significant improvement in the verbal and total intellectual coefficient, language capacity, and memory in girl children as compared to control (0.0% DHA) [77]. A comparative study among the infants fed on breast milk, a preterm formula supplemented with LCPUFAs, or a traditional preterm formula without LCPUFAs, showed that preterm formulas with LCPUFAs improve the visual understanding and development of infants like those fed with maternal milk [78]. A large study involving 28 countries demonstrated that better mathematics test scores in children from low-income families are proportionate to the DHA levels in breastfed children’s performance was found superior to non-breastfed children from high-income families and/or increased spending on education [79].

DHA level in the blood is positively correlated to cognitive abilities and inversely associated with cognitive decline. Activation of membrane GPR40 receptor by LCPUFA is responsible for driving neurogenesis by modulating synaptic plasticity in the adult brain. Dysfunction of GPR40 receptors can produce lipotoxicity in brain endothelial cells and neurons [80]. LCPUFA mediate GPR40 signaling modulates neurogenesis’s functional aspects, anti-nociceptive effects, anti-apoptotic effect, and Ca2+ homeostasis in Alzheimer’s disease (AD), and the nigrostriatal pathways. GPR40 is chosen as a potential drug target in treating several neuropathological disorders, including AD. During brain development, the DHA availability is also influenced by genetic polymorphisms of enzymes responsible for the endogenous conversion to LCPUFAs from their precursors.

Linoleic acid, 18:2n-6 (LA), and alpha-linolenic acid,18:3n-3 (ALA), are the two essential fatty acids. Imbalanced dietary intake of n-3 and n-6 fatty acids may lead to several neurological conditions. Brain concentrations of n-3 and n-6 PUFA derivatives are modulated by their dietary level and play a central role in regulating mood and cognition [81]. Endogenous abilities in the conversion of n-3 LCPUFAs from its precursor is affected by altered gene polymorphisms. The presence of gene-specific polymorphisms encodeΔ-5 and Δ-6 desaturases, enzymes involved in the synthesis of n-3 LCPUFAs from the precursor ALA, altered these fatty acid levels in particular DHA [82]. The f gene polymorphism (rs 174575) of Δ-6 desaturase enzyme in children allows higher DHA levels and better IQ test scores [83]. This indicates the importance of gene variations in the metabolism of n-3 LCPUFA and a consequential beneficial effect on brain development. Nine-month-old children supplemented with high DHA content showed significant cognitive performance and better arterial pressure at the end of infancy. N-3 LCPUFA supplementation, especially DHA, may favor optimum brain development that also has a protective effect against adult life’s cognitive and cardiovascular diseases [84]. Supplementation of DHA (400 mg/ daily) for 4 months significantly raised blood DHA levels, positively correlated with an increment of punctuation score obtained for vocabulary and comprehension tests in 4-year-old children [85]. Supplementation of children (*n* = 409, 3 to 13-year-old) in Australia, with 750 mg DHA and 60 mg EPA per day for 20 weeks, demonstrated a significant increment in children’s academic performance between 7 and 12 years old [86]. The supplementation of fish oil rich in DHA to 7–12-year-old ADHD children in Australia showed improved word reading, spelling capacity, and parents’ conduct. These changes were positively related to an increase in the DHA level of erythrocyte phospholipids [87].

Despite this evidence, absolute compliance with DHA supplementation of baby food has not been established yet. The Scientific Opinion of the European Food Safety Authority (EFSA) Panel (2014) expressed that *“…there is no convincing evidence that the addition of DHA to infant and follow-on formulae has benefits beyond infancy on any functional outcomes”*. The panel’s proposal about DHA supplementation of infant formula is based on DHA’s structural and functional roles in nervous tissue and retina and its exclusive presence in the brain and retina’s normal development.

### 2.2. Maternal DHA and Its Effects on the Placental Structure and Functional Development

Inappropriate structure and functional property of the placenta can impair the adequate exchange of nutrient and waste products between materno-fetal compartments during gestation [88]. The placenta’s transport efficiency critically determines the fetus’s optimal development and the well-being of the mother. Majorities of complicated pregnancies in humans are linked with the altered structure and function of the placenta. Inadequate supply of maternal blood to the fetus via the placenta is correlated adversity with risks of preeclampsia (PE), and intrauterine growth restriction (IUGR), preterm delivery (PT), etc. Shallow invasion of uterine spiral arteries by extravillous trophoblasts (EVTs) is a characteristics phenotype in PE, significant complications of the human pregnancy [89]. The EVTs are engaged in remodeling the uterine spiral artery to improve blood flow from the maternal circulation to the fetoplacental unit. Typically, humans’ trophoblast invasion is confined to the endometrium and the first third of the myometrium and restricted within the first trimester of pregnancy. The pathogenesis of PE is primarily caused due to inadequate angiogenesis and placental dysfunction, leading to adversity in pregnancy outcomes for both mother and babies. Angiogenesis forms a new blood vessel from pre-existing ones, critically important for placental development and vasculature [90]. Several growth factors are known to influence the angiogenesis processes that include vascular endothelial growth factor (VEGF), platelet-derived growth factor (PDGF), angiopoietin-like protein 4 (ANGPTL4), platelet-activating factor (PAF), matrix metalloproteinases (MMP), etc. [91].

Recent data highlights about DHA are that in addition to its essential requirement in the third trimester of pregnancy, it is also involved in the first trimester’s placentation processes. DHA stimulated tube-like formation in vitro to mimic angiogenesis during the early trimester of pregnancy in first-trimester human placental trophoblast cells, HTR8/SVneo cells [92,93]. Among the long-chain fatty acids tested in vitro, DHA was found the most potent stimulator of the in vitro angiogenesis in placental cells [92,94]. Moreover, DHA stimulated tube formation was partly mediated by increased expression and secretion of VEGFA synthesis, whereas oleic acid OA, ARA, EPA mediated their effects via increased synthesis of ANGPTL4 in these cells. The finding of n-3LCPUFAs on VEGFA synthesis and angiogenesis in placental cells opposed their other cell types’ actions [95,96]. The induction in the expression of VEGFA by DHA was found unique in many ways as its expression was induced by several growth factors and cytokines, including EGF, PDGF, TGFβ1, TNFα, and IL-1β, but not by any fatty acids. The mechanism of DHA’s action is still to be defined. However, DHA metabolites are unlikely to be involved as DHA induced VEGFA expression was not accompanied by COX-2 expression, a predominant mediator of DHA metabolites. Since peroxisome proliferator-activated receptor (PPAR,) agonist, and ligands failed to stimulate VEGFA expression, the involvement of PPARγ in DHA-stimulated VEGFA expression was unlikely. Both DHA and the EPA showed anti-angiogenic effects in cancer cells by inhibiting angiogenic mediators’ production, VEGF, nitric oxide (NO,) PDGF, COX-2, PGE2 [95,97]. EPA enhanced VEGF synthesis by activating GPR120 and PPARγ pathway in 3T3-L1 cells [98]. Although DHA metabolite, such as 4-hydroxy-docosahexaenoic acid (4-HDHA), inhibited PPARγ-mediated angiogenesis in endothelial cells [99], however, DHA metabolites are not involved in the tube formation and/ VEGF synthesis of the placental trophoblast cells as fatty acid receptor genes fatty acid-binding protein-4 (FABP4, GPR120, GPR40) and lipid metabolic genes (COX-2 and CAV-1) is not upregulated by DHA [94]. The precise mechanism of enhanced tube formation by EPA and DHA in placental trophoblast cells still to be ascertained. These LCPUFAs increased COX-2 messenger RNA mRNA expression, which may play a role in angiogenesis [94]. The mechanism that distinguishes DHA from other LCPUFA may be due to structural differences of the fatty acids. Further data postulates that DHA stimulates the expression of FABP4, an upstream target of VEGF in other cell systems. However, detailed work is essential to underscore DHA’s differential effects over other fatty acids in endothelial, tumor, and placental trophoblast cells.

DHA delivery is essential for fetal brain and eye development during the third trimester of pregnancy [93,100]. Due to the limited synthesis and endogenous DHA conversion, the growing fetus relies mostly on the maternal DHA’s placental supply [100]. Maternal plasma free fatty acids are taken up by the placental trophoblast via several membranes spanning proteins, such as fatty acid transport proteins (FAT/ cluster of differentiation 36 CD36, FATPs, and plasma membrane fatty acid-binding protein FABPpm) and intracellular fatty acid-binding proteins (FABPs) [101]. The mechanism of DHA uptake and transport in the placenta is not fully understood yet. However, a preferential uptake of DHA transport over other fatty acids via placental plasma membrane fatty acid-binding protein (p-FABPpm) was demonstrated [51,102,103]. Recently, a transporter major facilitator superfamily domain-containing 2A (MFSD2a) is present in the human placenta. MFSD2a) is located in the blood-brain barrier vessel and is involved in transporting the lysophosphatidylcholine form of DHA from the circulation into the brain. The role of MFSD2a in the placenta is not clear at this moment. Expression of the MFSD2a gene during pregnancy is used as a biomarker in predicting fetal neurodevelopment [104]. Maternal MFSD2a levels in blood were positively associated with the Z-score of head circumference at multiple time points at the neonate’s first year. Placental MFSD2a transporter expression decreased and correlated to decreased DHA in cord blood of women with gestational diabetes, indicating its role in contributing to materno-fetal DHA transport [51,105,106]. Not much is known about the cellular localization of MFSD2a in the human placenta yet, although its expression is altered in the placenta of gestational diabetes placenta and PE. It is still not known whether the expression of MFSD2a in normal pregnancy may be used as an indicator of DHA deficiency in the fetus’s brain.

Several researchers reported an increased incidence of preeclampsia in women with low n-3 fatty acids content in their red blood cells [107]. Increased n-3 LCPUFAs intake during pregnancy was beneficial for overall fetal growth and lowered the risk of early delivery and preeclampsia incidence [2,108]. In preterm birth, a positive association between placental DHA contents with placental weight was reported [109]. Maternal DHA content is, therefore, not only crucial for fetal growth and development but may also contribute a role in determining the placental size by stimulating first trimester trophoblasts mediated early placentation processes. International scientific experts recommend maternal consumption of pre-formed DHA for the prevention of premature birth [110], based on prevailing data from meta-analyses and extensive randomized controlled trial (RCT) studies [17,111]. The majority of the n-3LCPUFA supplementation trials were conducted during 16–20 weeks of gestation, well before the development of first-trimester pregnancy. Therefore, studies are required to evaluate the effects of n-3LCPUFA supplementation during pre-conception or before placentation prior to the 14th week of pregnancy to ensure optimal placentation and protect placental developmental-related abnormalities.

DHA incorporates a considerable amount of the fetal brain and retina during the third trimester of pregnancy [112,113], and this correlates with the development of normal eyesight and cognitive function [112]. DHA deficient condition can exist for an extended period after birth of very low birth weight (VLBW) babies since neonates miss the placenta’s DHA supply window due to shortened gestation time. Various studies demonstrated the beneficial effects of maternal intakes of n-3 fats during pregnancy in optimum growth and development of the brain and retina [114]. Maternal supplementation of n-3 LCPUFAs during pregnancy increased gestational duration with a slight increase in birth weight [115,116]. The improvement in birth weight and prolong gestation was confirmed by meta-analyses of several n-3 LCPUFAs supplementation studies during pregnancy [115]. Despite these, the DHA supplementation had mixed impacts on gestation length in the general population of pregnant women [74,117]. Low consumption of marine fish is found a decisive risk factor for preterm delivery [118]. Daily intake of n-3PCPUFA significantly lowered the rate of recurrent preterm delivery as concluded from a large multicentric clinical trial involving subjects with a history of preterm delivery [115]. The effects of n-3LCPUFA supplementation in high-risk pregnancy showed an inconsistent outcome [117]. Regular intake of n-3PCPUFA or placebo failed to prevent the recurrence of high-risk IUGR or pregnancy-induced hypertension [119]. The power and sample size can determine the measurable differences in the n-3LCPUFA supplementation trial’s birth outcome during pregnancy [17,113]. The improved birth weight due to n-3LCPUFA supplementation often arises due to the longer gestational length of these pregnancies [120]. The adequate intake of n-3 LCPUFA by the mother ensures increased availability of these fatty acids in the fetus [121]. Besides, post-natal supplementation of LCPUFAs also improved the subsequent development of vision and brain in VLBW infants [122].

### 2.3. Maternal Intakes of DHA and Its Impact on Fetal Brain Development

Despite studies supported the nutritional and metabolic requirement of n-3 PUFAs, but functional implications of the n-3 PUFA, like ALA, were not demonstrated. The first report on ALA deficiency was reported in 1982 when the parenteral formula containing a higher ALA (42.4% LA and 6.9% ALA) corrected the neurological disorders of a 6-years old girl [123]. Holman and coworkers then proposed that ALA was an essential fatty acid. The minimum requirement to prevent symptoms caused by ALA deficiency was in the range of 0.5–0.6% of total energy intake [123]. After consuming an ALA deficit diet, elderly patients developed dermatological disorders, particularly dermatitis, and flaky skin, together with deficient circulating EPA and DHA levels. The adverse skin symptoms were resolved, and plasma levels of EPA and DHA regained normal value once ALA was added to the formula [124]. Based on available evidence, a minimum intake of ALA and EPA plus DHA for an adult was fixed at 0.2–0.3 en% per day and 0.1–0.2 en% per day, respectively, indicating that in the absence of EPA and DHA, the endogenous biosynthesis of these fatty acids from ALA is significantly increased [125]. Considering data available to date and the substantial presence of DHA in the human nervous system, including in the brain and retina, it is agreed that humans can transform 1% of total ingested ALA into DHA [126]. The breast milk remained the only food supply to the neonate containing all the essential nutrients, including the necessary and obligatory n-3 and n-6 LCPUFAs, to ensure optimal brain development [127,128,129].

Colostrum levels of n-3 PUFAs are positively associated with infant mental development and [130]. In contrast, LA levels in colostrum are negatively associated with child cognitive scores at ages 2 and 3 years, independently of breastfeeding duration [131]. DHA’s relative content is ranged from 0.1% to 1% of the total fatty acids in human breast milk. The DHA content is vastly varied among populations globally, depending on their eating habits of fish or seafood intake or food cycle with land animals that feed on fish-meal or fish oils [43,132]. Over time, the level of DHA in breast milk is significantly decreased in the Western population, primarily due to lower intake of DHA containing foods [133]. According to the Food and Agriculture Organization (FAO), minimum dietary intake of 200 mg/day is suggested for pregnant women [134]; however, there is no universal consensus on the recommendation. However, the compliance was much better when pregnant women were advised or received dietary counseling about DHA’s physiological importance in pregnancy, as reflected in their increased consumption of n-3 LCPUFAs containing foods and/or supplements [135]. Dietary counseling about the benefits of marine fish or fish product consumption during pregnancy significantly boosted n-3 LCPUFAs [136]. FAO and many studies have established that benefit of consuming marine fish rich in n-3 LCPUFA quash the possible adverse effects of any heavy metal or other organic contaminants present [137]. Consumption of two portions of fish rich in DHA, such as salmon, tuna, anchovy, or mackerel, in a week may adequately comply with the minimum dietary requirements during pregnancy [138,139]. Low intake of marine foods that provide DHA during pregnancy is critical to obtain enough of these fatty acid levels, reflected in low DHA levels in umbilical blood [140]. Intake of *trans* fatty acid ingestion may also decrease the availability of n-3 LCPUFA to the mother and her child [141]. All these data suggest that the public health policies must be developed using DHA consumption for the population, especially pregnant and nursing women.

## 3. The DHA Uptake System in the Human Brain

Usually, non-esterified DHA is the major plasma pool for its supply to the brain [142,143]. DHA is also present as an esterified to lysophosphatidylcholine (LPC-DHA) pools in similar amounts. The brain maintains its fatty acid level via fatty acid uptake from circulation [144]. Astrocytes and endothelial cells of the blood-brain barrier (BBB) are significantly involved in the brain’s uptake of plasma fatty acids. The brain’s fatty acid uptake may occur via different mechanisms, such as passive diffusion and a saturable protein-mediated transport system.

**Figure 1:**
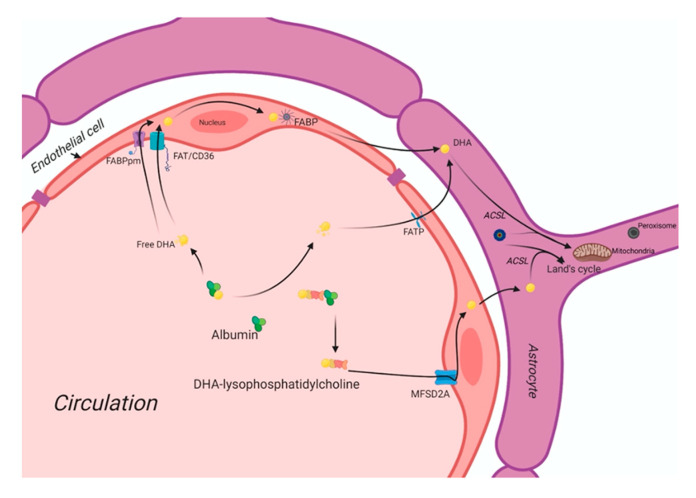
Docosahexaenoic acid (DHA) uptake system of the brain. DHA: Docosahexaenoic acid; MFSD2A: Major facilitator superfamily domain containing 2A; FABPpm: Plasma membrane fatty-acid binding proteins; FAT/CD36: Fatty acid translocase/cluster of differentiation 36; FABP: Fatty acid-binding protein; FATP: Fatty acid transporter protein; ACSL: long-chain acyl-CoA synthetase.

In circulation, free fatty acids (FFAs) are mainly bound by albumin. At the inner surface of endothelial cell membranes, a small fraction of FFAs is delivered into the subcellular compartments for further metabolism. At the same time, most FFAs diffuse into the cytosol with or without the help of the battery of cell membrane- and cytoplasmic fatty acid-binding proteins. Figure 1 shows the putative fatty acid transport system of the brain. There are four classes of fatty acid transport proteins involved in transportation in the adult brain, including fatty acid translocase (FAT/CD36), plasma membrane-fatty acid-binding proteins (FABPpm), fatty acid transport proteins (FATPs), and cytoplasmic FABPs. Additionally, MFSD2a is newly identified as a DHA transporter in the brain. Even though there is a preferential uptake by the brain of esterified DHA (LPC-DHA), this is not the major pathway by which DHA is transported into the brain. Despite being an abundant fatty acid in brain phospholipids, DHA cannot be synthesized de novo in the brain and must be imported across the blood-brain barrier, but its transportation pathways are unknown yet. MFSD2a is the major DHA transporter, is expressed exclusively in the endothelium of the BBB of micro-vessels. The MFSD2a-deficient mice brain had significantly reduced DHA concentrations with neuronal cell loss in the hippocampus and cerebellum. These mice also exhibited had microcephaly with severe cognitive deficits and anxiety. MFSD2a transports LPC-DHA, but not unesterified DHA, in a sodium-dependent manner. Notably, MFSD2a transports plasma pool LPCs carrying long-chain fatty acids (C>14). Long-chain acyl-CoA synthetases (ACSLs) are also involved in brain DHA uptake [51,102]. Brain development requires an incredible increase in the de novo synthesis and accretion of DHA, mediated by several factors, including sterol regulatory element-binding protein SREBP. In normal physiology, the activity of MFSD2a is regulated by SREBP to maintain a balance between de novo lipogenesis and exogenous uptake of LPC-DHA [145].

DHA is carried through the plasma by albumin and circulating lipoproteins. There are four classes of lipid transport proteins involved in DHA uptake of the brain that includes fatty acid translocase (FAT/CD36), plasma membrane fatty acid-transport proteins (FABPpm), and fatty acid transport proteins (FATPs) and cytoplasmic FABPs. MFSD2a, a specific protein, can transport plasma LPC-DHA, but not other DHA forms, across the blood-brain barrier to the neuron.

### DHA Deficiency during Fetal Brain Development and Its Impact on Cognitive Functions

During pregnancy and lactation, maternal intake of DHA ensures adequate maternal reserve deposited during pregnancy to support six-month breast-fed post-natal life. Maternal adipose resources during pregnancy cover the increasing demand for DHA in the early post-natal stage. Although it is difficult to estimate the quantity of DHA require in the diet for optimum brain development, the study from Kuiper et al. [146] first estimated the absolute requirement of DHA, ARA, and LA at 25 (conceptual age), 35 (preterm), and 45 (term) weeks. Based on the mother’s data fed on the western diet, the DHA accretion rate was found 42 mg/day in the last five weeks of pregnancy. The accretion of DHA was found double in the last five weeks of pregnancy compared with the first thirty-five weeks together. The DHA accretion rate largely determines the DHA deficiency during brain development in the brain during a term and preterm conditions. Thus, it is important to devise the DHA requirement for preterm babies based on the mother’s data from a different ethnic and dietary background. The majority of the available data suggests DHA’s optimal requirement during brain development in infants is fed on the Western diet. A scenario with high LA intake in the diet will further enhance the need for pre-formed DHA in infant formula. To fulfill enough maternal reserve of DHA, intake may be required well before conception.

In developing populations, where pregnant women’s usual diet is low in fats in which n-6 PUFAs are predominantly present with little intake of ALA or DHA as n-3PUFAs, most of the women start pregnancy with inadequate or insufficient n-3 PUFA status in their reserve. Under such a scenario, where a maternal reserve of DHA is low, endogenous synthesis of DHA from precursors is inadequate, resulting in an insufficient supply of DHA to the fetus that may affect DHA accretion in the brain during the brain development phase of the neonate. DHA’s placental deficiency can be correlated with the lower DHA accretion in the brain of the babies born preterm in the developing population. There is no clinical data available about DHA accretion in the brain and neonatal’s cognitive performance from the mother fed on the n-3 deficient diet in such a population. Therefore, optimal maternal intake of n-3 LCPUFAs is essential to fulfilling DHA’s neonatal requirement for the first six months. Maternal intake of DHA is correlated with the problem-solving skills of the children in some aspects. It was argued that inadequate intake of DHA could affect rapid accretion of DHA in the human brain, mainly when brain growth is maximal as with the third trimester or first six months of life. Adequate DHA during this period ensures the maturation of the prefrontal cortex’s specific brain domain that may support the problem-solving skills.

The impact of DHA deficiencies on cognitive functions is extensively studied using animal models in vivo. DHA deficiency can influence DHA’s endogenous synthesis in the brain and its impact on synaptic plasticity. A recent study with DHA deficient mice by silencing ELOVL2, an enzyme that is responsible for endogenous synthesis of DHA from its precursors, showed downregulation in the expression of neural plasticity factors (BDNF, Arc-1) with a concomitant increase in the pro-inflammatory markers (TNFα, IL-1β, inducible nitric oxide synthase iNOS) in the cerebral cortex of the DHA deficient mice [147]. The altered expression of these factors is also associated with the learning and memory function in the brain. The endogenous DHA deficient mice showed an alteration in microglial architecture and cytokine factors without involving astrocytes indicates that resident immune cells are affected in the brain by endogenous DHA deficiency. The replenishment with DHA restored the physiological expression of neuroinflammatory and neuroplasticity factors in the cerebral cortex. These data indicate that DHA plays a critical role in neuroimmune communication in brain function and synaptic plasticity.

The damaging effects of n-3 PUFA deficiency on brain lipid composition and memory performance were evidenced in lipopolysaccharides (LPS)-induced rat models, suggesting that in utero n-3 PUFAs deficiency could be a potential risk factor for neurodevelopmental disorders [148]. The n-3 PUFA deficiency in rats shows downregulated glutamate receptors and upregulated pro-inflammatory TNFα gene expression in the central nervous tissue independent of their effects on membrane composition [149]. Chronic deficiency of DHA over multiple generations during brain spurt affects the process of neurodevelopment by modulating the neuronal cell growth and differentiation, as well as neuronal signaling. The deficiency may cause a functional deficit in the offspring’s learning and cognitive efficiency by reducing intellectual potential and enhancing the risks of neurological diseases in adult life [150]. The n-3 fatty acid deficiency disrupts the peripheral balance of pro-and anti-inflammatory states in the brain due to altered systemic ARA: DHA ratio [151]. The excess ARA generates high prostaglandin concentrations, leukotriene, and thromboxane that lead to a pro-inflammatory state in the brain that disrupts the balance of anti- (n-3) and pro-inflammatory (n-6) eicosanoids due to alteration in GPR receptors’ signaling [152]. In the animal model, DHA’s maternal deficiency revealed reduced telencephalon structure in the hippocampus region [153]. Such a deficiency state affects region-specific brain development areas where the cerebral frontal cortex region is affected mostly, leading to hyper motor activity, reduced learning ability, and altered monoamine transmission [154]. The maternal DHA deficiency affects the offspring’s brain development in a gender-specific manner due to differential efficiency of endogenous DHA converting enzyme in males and females.

As the endogenous conversion of DHA from its precursor is more efficient in female newborns due to estrogen presence, the infant male is more susceptible to the risk of brain disorders, such as ADHD, Autism, etc., in their later life [155]. The maternal DHA deficiency state profoundly affects behavioral change in feed intake, anxiety, and stress response in the offspring [156]. The n-3 deficiency state triggers sucrose-motivated food intake preference due to alteration in the brain-rewarding pathway that may prompt children to consume calorie-dense foods [157]. Chronic deficiency of n-3 fatty acids for multiple generations induces anxiety-related stress behavior in the offspring due to altered expression of neuropeptide Y-1 receptor and glucocorticoid receptor in the pre-frontal and hippocampus of the rat brain [150,158]. Data from several in vivo studies suggest that DHA promotes neurogenesis by improving the membrane fluidity in the structural domain of the hippocampus, prefrontal cortex, and hypothalamus region to stabilize the neurodevelopment circuitry network required for learning and memory recognition processes [159]. N-3 fatty acid deficiency during utero development and in the postnatal state negatively impacts cognitive abilities [74,160]. DHA’s dietary deficiency increases the risk for neurocognitive disorders, whereas a diet enriched with DHA increases learning and memory and protects against cognitive decline during aging. However, whether increased intake of DHA can prevent the risk of brain disorders requires further investigation.

## 4. Maternal DHA Supplementation and Brain Development

During pregnancy, DHA’s importance for fetal brain development has been shown in a large observational study (*N* = 11,875). The study found that children born to mothers with a higher intake of seafood during pregnancy improved fine motor skills, more significant pro-social behavior, higher verbal intelligence, and higher social development scores at eight years of age [66]. A recent longitudinal cohort concludes that higher DHA status during pregnancy and lactation is associated with an infant’s problem-solving skills at 12 months [161]. But Crozier did not find any relationship between DHA level during pregnancy and cognitive performance of 4- or 6-year-old children [161].

A randomized controlled trial (RCT) found that taking 200 mg DHA orally daily for 4 months after delivery caused children’s higher cognitive abilities at 5 years of age [162]. Ogundipe et al. showed that 300 mg/day DHA supplementation on the last trimester of pregnancy correlates positively with an infant’s brain volumes (on MRI scan) [163]. DHA (600 mg/day) supplementation from 14.5 weeks of pregnancy until the delivery on KUDOS study found improved visual attention in infancy but no consistent long-term benefit in childhood [164]. DHA (120 mg/day) supplementation, along with EPA (180 mg/day), from the 20th week of pregnancy until the 30th day of the postpartum period among 18–35 years older women in Iran found primary neurodevelopment improvement among 4 to 6 months old children [165]. However, another RCT from Australia did not find any effect among the toddlers at 18 months of age after an 800 mg DHA intake in 2399 pregnant women during <21st weeks of gestation to delivery [17]. Maternal supplementation of DHA (400 mg/day) during pregnancy positively reflected the child’s (5–6 years) performance on language skills and short-term memory [166]. The positive effect of maternal DHA intake during gestation was observed in 18-month-old infants but not when they were 5 years old. DHA’s long-term effects may be too small to detect, or it is possible that the DHA intake was not good enough during gestation to have lasting effects.

A considerable DHA accretion occurs during the brain growth spurt beginning in the third trimester. However, there is no adequate information on whether DHA deficiency during early gestation can impact fetal brain development in the third trimester. Most DHA intervention studies on neurodevelopment were carried out from the second trimester of pregnancy. Therefore, the impacts of DHA intervention at 16 weeks of gestation may be too late as DHA is required during the first trimester for early placental growth and development, the critical step for future placenta’s adequate roles for the maternal supply of DHA for the fetal brain development. The inverse relationship of child Beery scores with maternal erythrocytes 22:4*n*-6 and 22: 5*n*-6 suggests that visual-motor integration development is compromised at low prenatal DHA. This is consistent with the time course of brain maturation; maturation occurs in the visual cortex before the prefrontal cortex.

Consuming DHA-rich eggs (135 mg DHA/egg) during pregnancy showed higher erythrocyte and umbilical cord DHA levels as compared with those pregnant women who consumed non-enriched-eggs (18 mg DHA/egg) [167]. Babies born to mothers who consumed DHA-enriched cereal-bars (300 mg DHA/bar) had increased visual acuity until four months of postpartum [168], and a better capacity to resolve problems with an improved organization of their dream [169]. Despite the beneficial effects of fish oil n-3 fatty acids in fetal development, recommendations to increase fish consumption for pregnant women are often met by the fear of heavy metal contaminations in these foods. However, many health professionals recommend avoiding or reducing fish consumption, especially for pregnant women. Despite some organoleptic problems, because the reconstituted product developed some uncomfortable smell that is not accepted for some mothers, the formula has proved to increase the DHA content of breast milk [170]. In Chile, a newly developed inexpensive formula overcame this organoleptic limitation and increased DHA availability for pregnant and nursing population [171].

Available data based on randomized controlled trials suggest beneficial effects of maternal supplementation of DHA on neonatal growth and cognitive development (Table 1). Several RCTs found that DHA supplementation on term and pre-term infants found a significant outcome on visual development during infancy [172]. Table 2 presents RCTs from the postnatal supplementation of DHA on the neonatal visual, verbal, and cognitive development. A meta-analysis of RCTs on routinely supplemented infant formula milk with DHA has found no beneficial role in neurodevelopmental outcomes [173]. A large trial (DHANI) was carried out in India, where prenatal and 6 months of post-partum 400 mg/day maternal DHA supplementation effects measure to evaluate the neurodevelopment outcome [174,175]. The study reported that the mean development quotient (DQ) scores in the DHA and placebo groups were not statistically significant after 12 months of mothers’ supplementation through pregnancy and lactation with 400 mg/d DHA [175].

**Figure 2:**
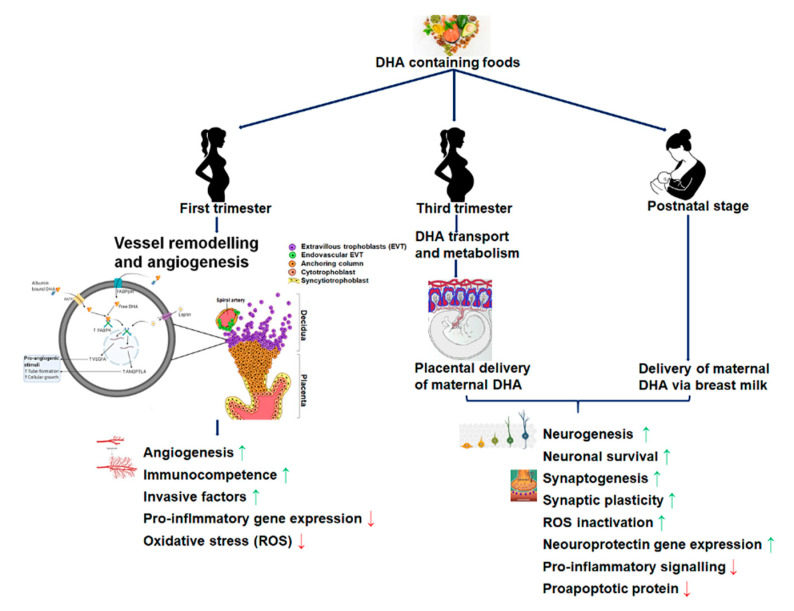
Maternal source and delivery of DHA at different stages of development affect fetoplacental and fetal brain development.

Figure 2 shows the accretion of DHA at different development stages that affect fetoplacental and fetal brain development. DHA accumulates substantially in the retina and cerebral cortex during the last trimester and the second year of life. Intervention studies have shown that improving maternal DHA nutrition reduces the risk of visual and neural development in infants and children. Several pieces of evidence supports the notion that maternal transfer of DHA to the infant before and after birth, with short and long-term, modulates neural functions. However, genetic variation responsible for endogenous conversion of DHA by fatty acid desaturases also influences essential fatty acid metabolism and may affect individuals’ optimal DHA requirements. Consideration of adequate DHA intake includes brain development, a balanced intake of n-3, and n-6 PUFAs in gestation, and lactation, and optimal fatty acid nutrition during pregnancy is required for infant neurodevelopment. Premature infants have a deficit in DHA shortly after delivery for several reasons, including missing the third trimester DHA accretion. More studies are required to assess DHA’s optimal dosage, delivery method, and duration of supplementation to evaluate DHA intake in premature infants.

DHA plays different roles in the gestation specific development of the feto-placental unit. During the first trimester, DHA possibly involves in decidual remodeling to establish early placentation by promoting the expression and secretion of angiogenic growth factors, such as VEGFA, FABP4, ANGPTL4, leptin, etc. DHA and its metabolites improve maternal-fetal immunocompetence by maintaining the oxidative stress, production of reactive oxygen species (ROS), and pro-anti-inflammatory balance maternal-fetal interface. DHA is delivered to the fetal brain during the third trimester via the placenta and subsequently via breast milk. In addition to DHA’s presence in the neonatal brain matter’s structural skeleton, mounting evidence suggests DHA helps several brain development processes, including neurogenesis, synaptogenesis, brain plasticity, inflammatory signaling, neuroprotection, etc.

## 5. Conclusions

The relationships between DHA on the brain development and function have been extensively studied. Under its ability to control membrane fluidity, the DHA also modulates neuronal density, neurotransmitter concentration, and synaptic activity by regulating the brain’s neuroinflammatory state. Increasing evidence suggests that DHA, the foremost important n-3 long-chain fatty acid in the brain, has neurotrophic and neuroprotective properties. Dietary n-3 PUFA deficiency during early development decreased the brain n-3 PUFA levels. Together, this body of evidence supports the proposition that DHA deficiency in utero increases the vulnerability of brain development. Most evidence indicates that the DHA accumulation is mainly influenced by dietary intake, specifically of preformed DHA. Insufficient intake of n-3 PUFA may lead to DHA deficiency states that could affect the offspring’s metabolic phenotypes by altering placental structure and function, fetal adiposity, body fat distribution, energy utilization, musculoskeletal growth, signaling between brain-adipose, epigenome stability, and inflammation [10].

## Figures and Tables

**Table 1:** Supplementation of DHA on the neonatal growth and cognitive development: a consolidated Randomized Controlled Trial (RCT).

Subject, Sample Size, Location	Dosages, Duration	Primary Outcome	References
Pregnant women, *n* = 350, USA	DHA 600 mg per day, <20 wk to delivery	Gestational duration ↑ Birth size ↑	Carlson et al., 2013 [45]
Pregnant women, *n* = 315, Germany, and others	DHA 500 mg and EPA 150 mg per day, <20 wk to delivery	Visual coordination 2.5 yr. children ↑ Cognitive development 5.5 yr children↑	Dunstan et al., 2008 Escolano et al., 2011 [52]
Pregnant women, *n* = 300, UK	DHA 300 mg, EPA 42 mg, ARA 8.4 mg per day for 12 wks from the third trimester	MRI of infant (*n* = 86) at birth show a correlation with DHA and brain volume ↑	Ogundipe et al., 2018 [163]
Pregnant women, *n* = 271, Canada	DHA 400 mg per day, 16 wk to delivery	Maternal DHA correlates with language and short-term memory development of 5.79 yr children ↑	Mulder et al., 2018 [166]
Pregnant women, *n* = 1094, Mexico	DHA 400 mg per day, 18–22 wk to delivery	Birth size and head circumference at birth ↑ The attention of 5 yr pre-school children↑	Ramkrishnan et al., 2010 [113]
Pregnant women, *n* = 2399, Australia	DHA 800 mg per day, <21 wk to delivery	No effects on cognitive and language development in 1.2 yr infant	Makrides et al., 2010 [17]
Pregnant women, *n* = 301, USA	DHA 600 mg per day, 14.5 wk to delivery	Cognitive behavior 10 mo to 6 yr; Visual attention ↑ No long-term beneficial effects	Colombo et al., 2019 [164]
Pregnant women, *n* = 143, Norway	DHA 1183 mg and EPA 803 mg per 10 mL per day, 18 wk to post-delivery 3 mo	Mental processing score at 4 and 7 yr age ↑ No effects on BMI at 7 yr age	Helland et al., 2003 [65]
Pregnant women, *n* = 150, Iran	DHA 120 mg and EPA 180 mg per day, 20 wk to post-delivery 1 mo.	Primary neurodevelopment outcome of 4–6 mo Infant ↑	Ostadrahim et al., 2018 [165]
Pregnant women, *n* = 98, Australia	DHA 2200 mg and EPA 1100 mg per day, 20 wk to delivery	Visual and coordination 2.5 yr children ↑	Dunstan et al., 2008 [52]
Pregnant women, *n* = 30, USA	DHA 214 mg as a functional food, 24 wk to delivery	Visual acuity 4 mo infant ↑	Judge et al., 2007 [168]

Abbreviations: DHA: Docosahexaenoic acid; ARA: Arachidonic acid; EPA: Eicosapentaenoic acid; Mo: Month; Yr: Year; Wk: Week; MRI: Magnetic resonance imaging. ↑ denotes “increased”.

**Table 2:** Supplementation of DHA on the neonatal visual, verbal, and cognitive development: a collection of Randomized Controlled Trials (RCTs).

Subject, Sample Size, Location	Dosages, Duration	Measured Outcome	References
Term formula-fed infant, *n* = 343, USA	DHA (0.32%–0.96%), ARA (0.64%) from 1–9 day to 1 yr	DHA (0.32%) group visual acuity ↑	Birch et al., 2010 [73]
Term infant, *n* = 420, Australia	DHA 250 mg and EPA 60 mg per day, birth to 6 mo	Accretion of DHA ↑ Early development of language and communication skills ↑	Meldrum et al., 2012 [76]
Infant BW < 1.5 kg, *n* = 141, Norway	DHA 32 mg, ARA 31 mg per 100 mL human milk per day, 1 to 9 wk after birth	Memory recognition and problem-solving skills of 6 mo infant ↑	Henriksen et al., 2008 [74]
Pre-term infant, *n* = 107, UK	DHA (0.5%) in the supplemented formula from birth to 9 mo	Verbal and intellectual coefficient of 9 yr girl ↑	Isaacs et al., 2011 [77]
Pre-school healthy children, *n* = 175, USA	DHA 400 mg per day for 4 mo of 4-yr-old children	Higher blood DHA correlates comprehension, punctuation, and vocabulary abilities ↑	Ryan et al., 2008 [85]
Indigenous school children three to13 yr, *n* = 409, Australia	DHA 750 mg, GLA 60 mg per day for 20 school week	Scholar performance in 7–12 yr children ↑	Parletta et al., 2013 [86]
Term infant, *n* = 227, USA	Algal DHA 200 mg per day for 4 mo after delivery	Cognitive abilities in 5 yr children ↑	Jensen et al., 2010 [162]
Pre-term infant, *n* = 361, USA	Algal DHA oil (17 mg/100 kcal), fungal ARA oil (34 mg/100 mL) from birth to 4 mo	Growth and development of pre-term infant till 118 wk ↑	Clandinin et al., 2005 [176]
Pre-term 23–24 wk infant, *n* = 90, USA	Algal DHA 50 mg per day from 1 to 6–7 wk at discharge	DHA levels of pre-term infant comparable to term placebo ↑	Baack et Al, 2016 [177]
ADHD 7–12 yr children, *n* = 90, Australia	DHA 1032 mg or 108 mg, EPA 264 mg or 1109 mg for 4 mo	Reading and spelling correlated DHA levels in the blood ↑	Milte et Al, 2012 [87]
Larger pre-term 30–37 wk, BW > 2 kg, *n* = 27, Taiwan	DHA (0.05%), ARA(0.1%) infant formula for 6 mo after birth	Mental development index at 6–12 mo ↑	Fang et Al, 2005 [75]
Breastfed 6 mo infant, *n* = 25–26, USA	DHA 115 mg/100 g baby food from 6 mo to 12 mo of the breastfed infant	Maturation of retina and visual cortex at 12 mo ↑	Hoffman et al., 204 [69]

↑ denotes “increased”.
